# RP-HPLC Method Development and Its Validation for Quantitative Determination of Rimonabant in Human Plasma

**DOI:** 10.1155/2012/625979

**Published:** 2012-04-23

**Authors:** Shravan Bankey, Ganesh Tapadiya, Jasvant Lamale, Deepti Jain, Shweta Saboo, S. S. Khadabadi

**Affiliations:** ^1^Department of Pharmaceutical Chemistry, Rajiv Gandhi Prodhyugiki Mahavidhyalaya, Bhopal 462036, India; ^2^Department of Pharmacognosy, R. C. Patel Institute of Pharmaceutical Education & Research, Shirpur 425405, India; ^3^Department of Pharmaceutical Chemistry, Rajiv Gandhi Prodhyugiki Vishwavidhyalaya, Bhopal 462036, India; ^4^Department of Pharmacognosy, Government College of Pharmacy, Osmanpura, Aurangabad 444604, India

## Abstract

A simple, accurate, and precise HPLC method was developed and validated for determination of rimonabant in human plasma. Following liquid-liquid extraction, chromatographic separation was accomplished using C18 column with mobile phase consisting of acetonitrile : water (90 : 10, v/v), drug was detected at 260 nm using UVdetector. The LOD and LOQ were 3.0 and 10.0 **μ**g/L, respectively. The method is linear in the interval 50.0–1000.0 **μ**g/L. The average extraction recovery of drug from plasma was found to be 92.2%. The percent CV of the method was found to be less than 10.8%, and accuracy was found between 94.5 and 106.7%. The assay may be applied to a pharmacokinetic and bioequivalence study of rimonabant.

## 1. Introduction

**Figure 1:**
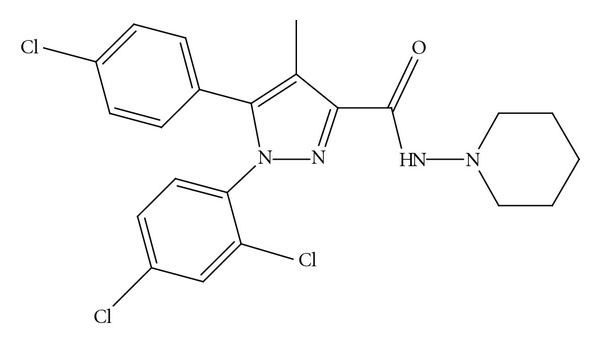
Structure of rimonabant.

Rimonabant (5-(4-chlorophenyl)-1-(2, 4-dichloro-phenyl)-4-methyl-N-(piperidin-1-yl)-1H-pyrazole-3-carboxamide, Figure 1) is a neurokinin-3 antagonist and selective cannabinoid (CB1) receptor antagonist used for the management of obesity [1, 2]. Rimonabant reduces the food intake and increases the energy expenditure. These effects are due to inhibition of the CB receptors situated in the mesolimbic area. They modulate the neurochemical activation of hypothalamic neurons and the state of relative energy balance. Rimonabant also inhibits the enzymes involved in lipogenesis [3]. Rimonabant has good oral bioavailability and long duration of action (8 hours). The half-life varies in healthy and obese individuals. In healthy adults with a body mass index of 18 to 28 kg/m^2^ receiving once-daily doses of rimonabant 20 mg, the half-life ranged from 6 to 9 hours. In obese individuals with a body mass index of >30 kg/m^2^, the half-life was longer (16 hours), due to the larger peripheral volume of distribution. Following multiple once-daily doses of 20 mg to healthy subjects in the fasted state, maximum plasma concentrations of rimonabant are achieved in approximately 2 hours, with steady state plasma levels achieved within 13 days (C  max⁡ = 196 ± 28.1 *μ*g/L; AUC 0–24 = 2960 ± 268 *μ*g·h/L) [4]. Rimonabant appears to increase the risk of suicidality and was not approved by FDA. The EMEA (European Medicines Agency) has recommended suspending the approval in Europe but the drug may be remerged in tobacco and smoking-cessation therapies. There were few LC-MS/MS method available for the determination of rimonabant [5, 6]. These methods may not be widely accessible due to their cost and ion suppression/enhancement effects, which may require expensive sample and clean-up procedures. Here is a developed economical method which can be used for the determination of rimonabant from plasma.

## 2. Experimental

### 2.1. Materials and Reagents

Rimonabant API was supplied by Cadila Healthcare Limited, Ahmedabad (India). Acetonitrile (HPLC grade) was purchased from Qualigens Fine Chemicals, India. Methanol (HPLC grade) was purchased from Rankem, RFCL Limited (India). Water was purified using a Milli-Q system (YoungLin Basic 370 series).

### 2.2. Apparatus and Chromatographic Conditions

HPLC analysis was performed on YoungLin system equipped with quaternary SP930D gradient pump, a vacuum degasser and mixer, a UV730D UV/VIS detector, and a rheodyne injector holding 20 *μ*L loop. The signals were acquired and analyzed using Windows XP-based YoungLin Autochro-3000 software. The separation of the compound (rimonabant) was made on a Nucleosil-C18 column (250 mn × 4.6 mm, 5 *μ*m particle size) using isocratic elution. The flow rate was 1 mL/min. UV detection was performed at 260 nm using mobile phase acetonitrile and water (90 : 10, v/v). Peak identity was confirmed by retention time (6.67 min).

### 2.3. Preparation of Stock Solutions, Calibration Standard, and Quality Control Samples

The standard stock solution of rimonabant (1.0 mg/mL) was prepared in acetonitrile. The working standard solution (10 *μ*g/mL) was prepared by diluting the stock solution in acetonitrile. A serial calibration line of samples of concentration of 50, 100, 200, 300, 400, 500, and 1000 *μ*g/L of rimonabant was prepared by diluting definite aliquots of working standard with plasma up to 2 mL and then precipitated with 3 mL of acetonitrile, followed by centrifugation at 5000 g at room temperature for 16 min, and the supernatant was filtered with 0.22 *μ*m filter. The quality control samples (low, medium, and high) were also prepared by diluting standard solution with plasma to form concentrations of 150, 450, and 800 *μ*g/L of rimonabant. A calibration curve was made from a blank sample (a drug-free plasma sample) and seven plasma-spiked samples with drug covering the total range (50–1000 *μ*g/L). Such calibration curves were generated on three consecutive days.

### 2.4. Specificity

Specificity of the method established by using six different lots of plasma samples (4 normal lots, one haemolised, and one lipemic lot). In each plasma lot, blank samples and LLOQ (lower limit of quantitation) were processed and analysed as per assay procedure to determine any significant interference at the RT of analyte.

### 2.5. Sensitivity

The limit of detection and limit of quantification were determined by calculating the S/N method, for this LLOQ (lower limit of quantitation 50 *μ*g/L) is diluted with plasma by serial dilution (50.0, 25.0, 10.0, 5.0, 3.0, 2.0, 1.0 *μ*g/L), processed and analysed in replicate sets with blank samples and the % CV of the areas obtained is calculated.

### 2.6. Extraction Recovery

**Figure 2:**
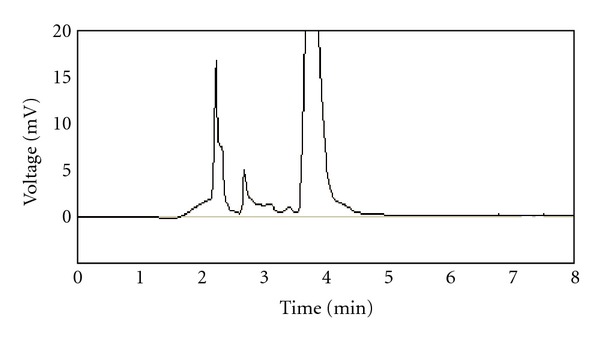
Chromatogram of blank plasma.

**Figure 3:**
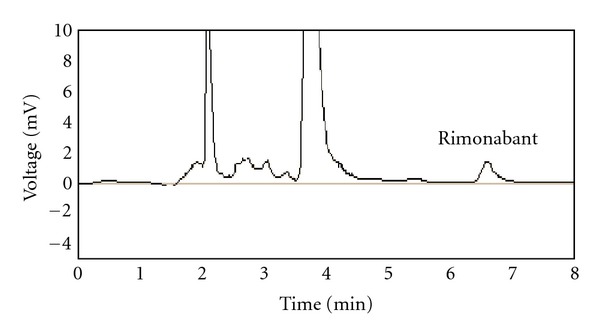
Chromatogram of low quality control sample.

Recovery of rimonabant was evaluated by comparing the mean peak areas of six-extracted low-quality control samples with the mean peak areas of six neat reference solutions containing the same amount of the test compound (Figures 2 and 3). 

### 2.7. Precision and Accuracy

The intrabatch, interbatch, interday, and analyst to analyst precision and accuracy of the developed method were evaluated in plasma samples spiked with rimonabant at each QC level. Intrabatch and interbatch precisions and accuracies, and the samples that had been spiked at a concentration of 150.0, 450.0, and 800.0 *μ*g/L were assayed. The interday precision and accuracy were evaluated on three consecutive days and two different analysts evaluated analyst-to-analyst precision.

### 2.8. Stability

The low QC and high QC samples (150 and 800 *μ*g/L) treated as sample preparation was kept at room temperature for 24 hours and then the stability was determined. The freeze-thaw stability was determined after three repeated freezing and thawing cycles on day 0, 1, and 2. For each concentration and each storage condition, six replicates were analyzed in one analytical batch. The concentration of rimonabant after each storage period was related to the initial concentration as determined for the samples.

## 3. Results and Discussion

### 3.1. Development of LC Method

For optimum detection and quantitation of rimonabant in human plasma by liquid chromatography, it was necessary to maintain the chromatographic condition throughout the experimentation. The effect of acetonitrile content in the acetonitrile/water mobile phase on rimonabant retention time was investigated. As expected in reversed-phase HPLC, increasing the water content from 10 to 20% (v/v) resulted in a general increase in retention time due to greater hydrophobic interaction between the bonded alkyl stationary phase and the drug. A mobile phase composed of acetonitrile water in a ratio of 90 : 10 (v/v) at flow rate 1 mL/min was used to complete separation and detection of rimonabant by UV detector at relatively low concentrations without interference of sample matrixes. The high percentage of acetonitrile in the mobile phase allows rapid determination of drug with retention time less than 7 min. Figures 2 and 3 show the representative chromatograms of blank plasma and plasma spiked with rimonabant at 0.050 *μ*g/mL.

### 3.2. Validation of LC Method

#### 3.2.1. Specificity

Observed retention time for rimonabant was found to be 6.67 min. Plasma samples of different lots were found to be free from interfering substances at the retention time of rimonabant, and there is no impact of haemolysis or lipemic plasma in peak detection.

#### 3.2.2. Linearity, Limit of Detection, and Limit of Quantification

The most variable regression equation of calibration curve was *y* = 0.0564*x* − 0.0318 (*r* ^2^ = 0.9992) and the acceptable minimum criterion of the correlation coefficient (*r*) must be = 0.99 [7], hence the correlation coefficient showed good linearity. The limit of detection (LOD) and limit of quantitation (LOQ) of rimonabant in plasma samples were determined to 3.0 and 10 *μ*g/L, respectively. The calibration curve range is suitable to measure plasma concentration profile of the formulations, since it is covering well enough the C_max⁡  range_ (192 ± 28 *μ*g/L) reported in the literature [4].

#### 3.2.3. Inaccuracy and Imprecision

A total of five batches were tested for the experiments, and percent of CV and inaccuracy ranged from 4.3 to 5.1 and 97 to 101.9, respectively. Intra-day, interbatch, interday, and analyst-to-analyst imprecision showed a percent CV of 3.2 to 4.0, 3.4 to 3.7, 2.7 to 5.1, and 2.1 to 6.4%, respectively. The inaccuracy of proposed method was found between 94.5 to 106.7%. The data proved good precision of the developed method. The results of intrabatch, interbatch, interday, and analyst to analyst are illustrated in Table 1.

#### 3.2.4. Extraction Recovery

Protein precipitation was employed for the extraction of drugs from biological matrix, that is, plasma, and acetonitrile is used as the precipitating solvent because it shows the good recovery of drug from the plasma (Table 2). Extraction of drug from plasma was also tried with other organic solvents as precipitating agent, namely, methanol and salt solutions (sodium sulphite) of different concentrations (12.6, 15.8, and 21%). The recovery with methanol is comparable to acetonitrile, but 3 mL of methanol is needed for complete precipitation of 1 mL of plasma, which diluted the drug concentration in sample to half as compared to acetonitrile. In case of salt solutions as precipitating agent till 21% salt concentration followed by centrifugation at 10000 RPM for half an hour, clear supernatant was not observed. Therefore, acetonitrile was selected for extraction as it shows highest extraction recovery with minimum dilution factor. 

#### 3.2.5. Stability

Twenty-four-hour room-temperature storage and freeze-thaw cycles for low- and high-quality control samples indicated that rimonabant was stable in human plasma under experimental condition. Stability results are illustrated in Table 3.

## 4. Conclusion

The HPLC method for determination of rimonabant in human plasma has been developed. Method validation has been demonstrated by a variety of tests for specificity, sensitivity, linearity, precision, accuracy, recovery, and stability. The validated interval (50.0–1000.0 *μ*g/L) is sufficient to measure plasma concentration profile of the formulations having a dose of 20 mg in bioequivalence study obtained. The result of analysis suggests the applicability, reproducibility, and utility of the method for direct estimation of rimonabant in plasma sample.

## Figures and Tables

**Table 1:** Imprecision and inaccuracy results of validation.

Spiked conc. (*μ*g/L)	Conc. found mean ± SD (*μ*g/L)	CV (%)	Accuracy (%)
Intraday imprecision and inaccuracy (*n* = 18)			
150	151.8 ± 6.00	4.0	101.2
450	442.3 ± 19.51	4.4	98.3
800	780.6 ± 25.30	3.2	97.6
Interbatch imprecision and inaccuracy (*n* = 12)			
150	153.9 ± 5.20	3.4	102.6
450	430.5 ± 16.70	3.9	95.7
800	810.6 ± 30.3	3.7	101.3
Interday imprecision and inaccuracy (*n* = 12)			
150.0	156.4 ± 8.04	5.1	104.3
450.0	435.2 ± 17.90	4.1	96.7
800.0	756.2 ± 20.45	2.7	94.5
Analyst to analyst imprecision and inaccuracy (*n* = 18)			
150.0	160.0 ± 10.30	6.4	106.7
450.0	458.3 ± 9.80	2.1	101.8
800.0	780.2 ± 24.90	3.2	97.5
Global imprecision and inaccuracy (*n* = 30)			
150.0	152.9 ± 6.60	4.3	101.9
450.0	436.4 ± 22.05	5.1	97.0
800.0	795.6 ± 36.59	4.6	99.5

**Table 2:** Extraction recovery of rimonabant from plasma (*n* = 6).

Analyte	Conc. added (*μ*g/L)	Mean recovery ± SD (%)	CV (%)
Rimonabant	150.0	93.9 ± 12.00	8.5
	450.0	91.3 ± 22.20	5.4
	800.0	91.3 ± 14.60	2.0

The average global recovery = 92.2%, and percent CV of global recoveries = 1.6%.

**Table 3:** Bench top and freeze-thaw stability of rimonabant (*n* = 6).

Nominal conc. (*μ*g/L)	Mean found conc. (*μ*g/L)			CV (%)	CV (%)
Bench top stability	0 hrs	12 hrs	24 hrs	12 hrs	24 hrs
150.0	155.2	144.5	135.8	7.9	10.8
800.0	785.3	767.6	760.9	1.0	1.6
Freeze-thaw stability	Day 0	Day 1	Day 2	CV day (%) 1	CV day (%) 2
150.0	149.5	147.4	143.2	4.6	5.4
800.0	789.9	795.6	789.1	0.5	1.4
